# Electrophysiological damage to neuronal membrane alters ephaptic entrainment

**DOI:** 10.1038/s41598-023-38738-x

**Published:** 2023-07-24

**Authors:** Gabriel Moreno Cunha, Gilberto Corso, Marcelo M. S. Lima, Gustavo Zampier dos Santos Lima

**Affiliations:** 1 Departamento de Física Teórica e Experimental, Universidade Federal do Rio Grande do Norte, Natal, RN 59078-970 Brazil; 2 Departamento de Biofísica e Farmacologia, Universidade Federal do Rio Grande do Norte, Natal, RN 59078-970 Brazil; 3 Departamento de Fisiologia, Universidade Federal do Paraná, Curitiba, PR 81531-980 Brazil; 4 Departamento de Farmacologia, Universidade Federal do Paraná, Curitiba, PR 81531-980 Brazil; 5 Escola de Ciências e Tecnologia, Universidade Federal do Rio Grande do Norte, Natal, RN 59078-970 Brazil

**Keywords:** Neurodegenerative diseases, Alzheimer's disease, Parkinson's disease, Mathematics and computing, Biological physics

## Abstract

The brain is commonly understood as a complex network system with a particular organization and topology that can result in specific electrophysiological patterns. Among all the dynamic elements resulting from the circuits of the brain’s network, ephapticity is a cellular communication mechanism that has received little attention. To understand the network’s properties of ephaptic entrainment, we start investigating the ephaptic effect on a single neuron. In this study, we used numerical simulations to examine the relationship between alterations in ephaptic neuronal entrainment and impaired electrophysiological properties of the neuronal membrane, which can occur via spike field coherence (SFC). This change in frequency band amplitude is observed in some neurodegenerative diseases, such as Parkinson’s or Alzheimer’s. To further investigate these phenomena, we proposed a damaged model based on the impairment of both the resistance of the ion channels and the capacitance of the lipid membrane. Therefore, we simulated ephaptic entrainment with the hybrid neural model quadratic integrate-and-fire ephaptic (QIF-E), which mimics an ephaptic entrainment generated by an LFP (simulate a neuronal group). Our results indicate a link between peak entrainment (ephapticity) preference and a shift in frequency band when damage occurs mainly in ion channels. Finally, we discuss possible relationships between ephaptic entrainment and neurodegenerative diseases associated with aging factors.

## Introduction

Neural oscillations are recognized as important mechanisms for understanding physiological and pathological phenomena^1,2^. Brain rhythms originate from cell communications^3^, by exchanging small molecules and ions, as gap junctions and synapses; or by electric fields^4,5^. Communication made by exclusively electric fields (short-range interactions)^4,5^ is called ephapticity^3,4,6,7^, and it is known for several decades^6,8^. However, its physiological function in the central nervous system is still poorly understood^3,4,9^. Despite the lack of information regarding the physiological function of ephapticity, some empirical studies have shown that ephaptic communication has an important role in synaptic plasticity^3,10^ or even in neural dysfunction^5,11^.

Clusters of neurons can exchange information by connecting with each other in particular rhythms, depending on the task involved^2,12–14^. Nevertheless, factors associated with aging in the nervous system can generate neural damage, and undermine dynamics of rhythms and consequently neuron communication^15–17^. In neurodegenerative diseases due to the aging process, such as Parkinson Disease (PD), Alzheimer Disease (AD) or Lewy body dementia (DLB), accumulations of peptide (oligomers) are observed^18–21^. As an example of dysfunctional proteins are the $\alpha$-synuclein ($\alpha$-syn in PD and DLB), that are associated with the formation of Lewy bodies, and $\beta$-amyloid (in AD without Lewy body’s), which make up the senile plaques^18–21^. Oligomers have toxic cellular effects, compromising the structure of the plasmolemma, either by altering lipid and cholesteric composition^18,22,23^, or by inactivating ion channels^15,23–26^. Therefore, the brain rhythms from the damage regions are amended^12,15,27^, as observed in PD by the increase in the amplitude of the $\beta$ band^28–30^; or the lack of synchrony (low amplitude) in $\alpha$ and $\beta$ bands and high synchronizations in $\delta$ and $\theta$ in AD^27,31,32^.

The idea that collective neuronal behavior is intrinsically related to individual neuron properties is a fundamental principle in understanding the dynamics of networks^1,33^. Although networks are composed of numerous interconnected neurons, every neuron has its own particular dynamics and behavior, which is associated with its inherent features, for instance, its intrinsic rhythms^34,35^. In this context and regarding the importance of applying mathematical models for neuronal studies^36–40^, the present work aims to investigate the hypothesis that given a level of impairment of the single neuronal membrane may produce a change in its ephaptic entrainment. To this purpose, we adopted the QIF-E hybrid model, which uses the electrophysiological properties of capacitance and electrical resistance of the membrane for the simulation^39,41^. Thus, two new parameters related to plasmolemma damage are thought for the modified QIF-E model: (1) The parameter *h*, associated with damage to the characteristics of the lipid bilayer; and (2) The *b* parameter, related to dysfunction of ion channel opening. As a result, for different degrees of damage in the electrophysiological parameters, a change in the frequency peak of the ephaptic entrainment is observed. Our results emphasize a relation between ephaptic communication in the process of frequency band preferences and neurodegenerative diseases.

The study begins with a description of the mathematical model used to simulate ephaptic entrainment. In the following, is shown the proposal for the mathematical development of the electrical damage parameters, and their applications in the hybrid model. The Spike Field Coherence (SFC) tool is explained as a way to quantify the entrainment. The results obtained are shown in section Results. Finally, a discussion and conclusion is made about the impacts of ephapticity on the emergence of frequency bands with the loss of neuronal membrane function in degenerative processes associated with senility diseases.

## Firing neuron model with dysfunctional membrane

**Figure 1:**
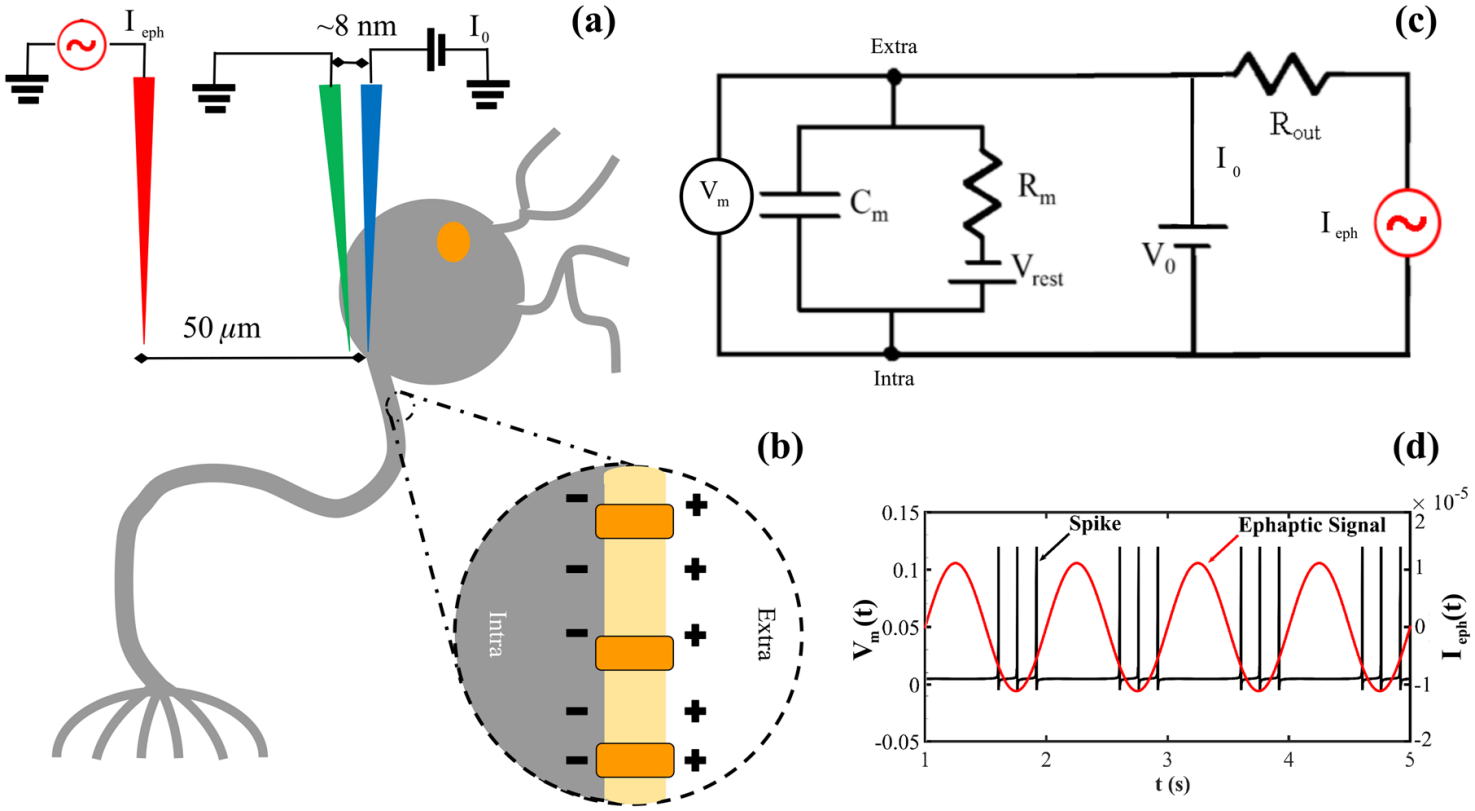
Ephaptic Entrainment (**a**) Representative scheme of the experiment made by Anastassiou, et al.^4^ for ephaptic entrainment, and here simulated by the QIF-E^41^ model. (**b**) Magnification of the structure of the plasmatic membrane (yellow) with the ion channels (orange) and the ionic separation promoted between the extra-(positively charged - white) and intracellular (negatively charged - gray) milieu. (**c**) Simplified equivalent electrical circuit for the ephaptic entrainment experiment. (**d**) Temporal series from QIF-E simulation for $V_{m}(t)$ (black) and $I_{eph}(t)$ (red).

The quadratic integrate-and-fire model with ephapticity (QIF-E)^41^ is a simplified neuron model and a type of integrate-and-fire neuron that describes action potentials in neurons inserted in an electric field, given by the LFP, which here is referred as the ephaptic term. In contrast to physiologically accurate but computationally expensive neuronal models, the QIF-E model only seeks to produce action potential-like patterns and ignores subtleties such as control variables. According to Cunha et al.^41^, the ephaptic communication can be simulated with the QIF-E hybrid model, datum by the equation:

1 $$\begin{aligned} \frac{dV_{m}(t)}{dt} \quad = \quad \underbrace{ \frac{(V_{m}(t)-V_{rest})(V_{m}(t)-V_{{thresh}})}{{C_{m} R_{m}}(V_{{thresh}} - V_{rest})}}_{{\textbf {Quadratic term}}} \quad - \quad \underbrace{ \frac{\rho _{ext} I_{eph}(t)}{4\pi {C_{m} R_{m}} r}}_{{\textbf {Ephaptic term}}} \quad + \quad \underbrace{ \frac{I_{0}}{{C_{m}}}}_{{\textbf {Synaptic term}}} , \end{aligned}$$

and, if $V_{m} \ge V_{peak}, V_{m} = c$. In Eq. (1), $C_m$ is the membrane capacitance, $V_m$ the membrane potential, $V_{rest}$ the rest potential, $V_{{thresh}}$ the threshold of excitation value, $R_m$ the membrane resistance and $I_{0}$ the synaptic current across the membrane. The *r* parameter in the equation of the QIF-E model, is the distance between the external stimulus electrode (red in Fig.1a) and the electrode corresponding to $V_{out}$ (green in Fig.1(a)). Finally, $I_{eph}$ is the ephaptic current that flow through of the membrane. The Table 1 shown the QIF-E model quantities use in the simulations. The ephaptic entrainment experiment are shown in the Fig.1, with the equivalents in the QIF-E model.

**Table 1:** Cell membrane biophysical parameters employed in the simulation of the quadratic integrate-and-fire model.

Quantity	Value	Description	References
$V_{rest}$	− 65 mV	Rest potential	^42,43^
$V_{{thresh}}$	− 55 mV	Excitation thresholds	^42,43^
$C_{m}$	$2.10^{-2} F/m^{2}$	Membrane capacitance	^44^
$V_{peak}$	+55 mV	Peak Value	^42^
c	− 70 mV	Hyperpolarization constant	^42^
$\rho _{ext}$	3.5 $\Omega$.m	Extracellular resistivity	^45,46^
*r*	50 $\mu$m	Distance between stimulus electrode and the position of $V_{out}$	^45^
$R_m$	$1.10^{-1} \Omega m^{2}$	Resistance of the neuronal membrane	^44^

**Figure 2:**
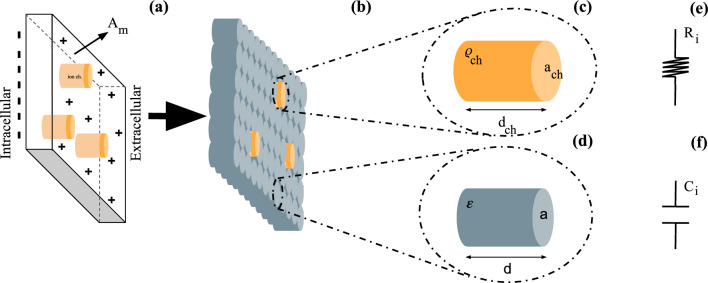
**Representation of the unity volume element of the cell membrane.** (**a**) Cell membrane (1) composed of various biological elements. (**b**) Membrane subdivided into equal volume elements. (**c**) Electrical properties of the ionic channel. (**d**) Electrical properties associated to unit capacitance of the membrane. (**e**) Equivalent resistor to an ionic channel present in the membrane. (**f**) Equivalent capacitance to a unit volume of the neuronal membrane.

In this work, we have built the hypothesis that deficits in parameters associated to maintenance of synaptic homeostasis could dramatically impact ephaptic communication. Hence, we developed an adaptation in QIF-E model, such that the electrical properties of the model can be changed, in order to simulate the damage in the membrane. For this purpose, the neuronal membrane (Fig.2a) was conceived like an ensemble of unitary elements (Fig.2b), with each individual volume equivalent to or an ionic channel (Fig.2c) as electric resistance (Fig.2e); or a phospholipid structure patch (Fig.2d) as capacitance (Fig.2f).

### Change in electric functionality of the generic ionic channels.

In accordance with the electrophysiological approach, the values of resistance were associated to the average values of the ionic channels. Therefore, the resistance of the membrane is provided by: $R_{m} = \rho \frac{d}{A}$. The $\rho$ parameter is the channel resistivity and reports the capacity of the channels to resist to ionic flows. The *d* distance is the length of the ionic channel. Finally, *A* is the total area (transversal sections sum) of available channels (open channels) to ionic flow through the neuronal membrane. For a damage analysis of neuronal membrane, we changed the $\rho = \rho _{ch}$ and the $d = d_{ch}$ and assign equal values of geometric and electrical properties to each unit of volume (See Fig.2b,c). The total area associated to the ionic channels can be described by $A = n.a_{ch}$; with *n* equal to the maximum ion channels number, and $a_{ch}$ is the single channel cross-section.

**Figure 3:**
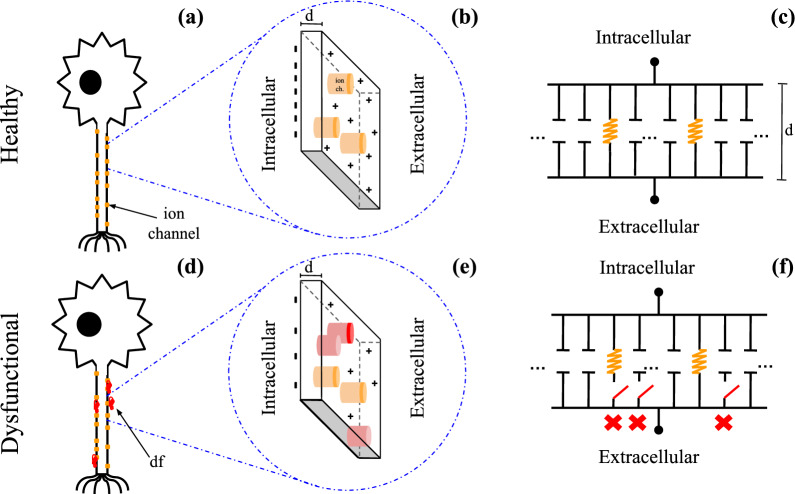
**Functional and Dysfunctional Scenarios.** (**a**) Schematic representation of healthy neuron and the ionic channels (orange). (**b**) Healthy neuronal membrane amplified, with the ionic channel (orange) (**c**) The neuronal membrane representation on the basis of its electrophysiological properties. Resistances and capacitance provide, by classical electromagnetism, a description of the membrane average performance. (**d**) The neuron representation in a dysfunctional scenario, described by a damage (red) along of axon. (**e**) Cellular membrane applied with damage occurrence (red), leading to ion channel inactivation (improve of resistance), or a decrease of membrane capacitance. This damage can breach the neuronal membrane, as in the PD case^23^. (**f**) Damage in the membrane electrophysiological properties caused by degeneration. Shown partitions of the resistance and capacitance opened in the circuit.

Total resistance of the membrane is provided by an association of unitary resistances, shown in Fig.3c. Thus:

2 $$\begin{aligned} \frac{1}{R_{m}} \quad = \quad \frac{1}{R_{1}} + \frac{1}{R_{2}} + ... + \frac{1}{R_{n}}\quad = \quad \frac{1}{ \frac{\rho _{ch} d_{ch}}{a_{ch}}} + \frac{1}{\frac{\rho _{ch} d_{ch}}{a_{ch}}} + ... + \frac{1}{\frac{\rho _{ch} d_{ch}}{a_{ch}}} \quad = \quad \frac{a_{ch}}{\rho _{ch} d_{ch}} + \frac{a_{ch}}{\rho _{ch} d_{ch}} + ... + \frac{a_{ch}}{\rho _{ch} d_{ch}} \end{aligned}$$

Then the resistance is described by:

3 $$\begin{aligned} \frac{1}{R_{m}} = n\frac{a_{ch}}{\rho _{ch} d_{ch}} \quad \rightarrow \quad R_m = \frac{\rho _{ch} d_{ch}}{n.a_{ch}} \end{aligned}$$

We idealized the dysfunctional scenario, for our model, as an inactivation of ion channels, leading to an increase in membrane resistance (See Fig.3f). The decrease of functional channels number will be analyzed by the difference between the total number of membrane channel, *n*, and the damage ionic channel parameter, *m*, i.e., $(n-m)$. Known that $n \in \mathbb {N}$ and $m \le n$, the general expression to the resistance of neuronal membrane to provide the dysfunctional situation description ($m \ne 0$) is:

4 $$\begin{aligned} {R_{dfl}} = \frac{\rho _{ch} d_{ch}}{(n-m)a_{ch}}. \end{aligned}$$

insulating the term: $\frac{\rho _{ch} d_{ch}}{a_{ch}}$ (related to ionic channel), in the Eqs. (3) and (4), we obtain:

5 $$\begin{aligned} (n-m){R_{dfl}} = n R_{m} \end{aligned}$$

Normalizing the expression by the total number of ion channels (*n*):

6 $$\begin{aligned} {R_{dfl}} = \frac{n}{(n-m)} R_{m} = \frac{1}{\frac{(n-m)}{n}} R_{m} = \frac{1}{(1-\frac{m}{n})} R_{m} = \frac{1}{(1-b)} R_{m} \end{aligned}$$

being $b = \frac{m}{n}$ the new parameter that describe the ion channel inactivated proportion (damaged).

With the progression of dysfunction, the proportion value increase of $b \approx 0$ (healthy) to the condition $b = 1$ (total damage), with values in $0 \le b < 1$. Thereby, the model can be expressed through the new parameter of ion channel damage.

### Change in the membrane capacitive functionality

The neuronal membrane phospholipid structure can be thought as a capacitor (Fig.2f), with the total capacitance provided by the expression: $C_{m} = \varepsilon _{m}\frac{A_{m}}{d}$. The $\varepsilon _{m}$ parameter is associated to the membrane electrical permittivity (standard); the distance *d*, to the neuronal membrane thickness and the area $A_{m}$ to the total area available for the ion accumulation in the membrane. Similarly to the ionic channel’s resistance, the membrane average capacitance can be associated to a sequence of unitary capacitors (Fig. 3c). The total area $A_{m}$ is split in equal units area (similar to ionic channels area). Therefore, the membrane total capacitance is provided by each capacitor unit sum ($C_{i}$): $C_m = C_1 + C_2 + C_3 +... + C_j$.

We consider that each capacitor unit are equivalents, this means that the element’s electric permittivity $\epsilon$, the area *a* and the membrane thickness *d*, are equal to each other, then $C_1 = C_2 = C_3 =... = C_j = C$. Therefore:

7 $$\begin{aligned} C_{m} = \sum ^{j}_{i=1} C_{i} = j C \end{aligned}$$

where *j* is the total number of healthy capacitors *C*. The loss of the membrane function is modeled by the damage in unity capacitors, computed through of the inactivated capacitors (committed), defined by *g* (See Fig.3g). Known that $g \in \mathbb {N}$ and $g \le j$:

8 $$\begin{aligned} { C_{dfl}} = (j-g) C \end{aligned}$$

Normalizing the above expression via a proportion of committed capacitors by the total number of capacitors $h = \frac{g}{j}$, we obtain:

9 $$\begin{aligned} {C_{dfl}} = j C (1-h) = C_m (1- h) \end{aligned}$$

with $C_{dfl}$ as the dysfunctional membrane capacitance.

In this way, it is possible to measure, via SFC, the changes in ephaptic communication with membrane damage progression: *b* and *h* (Fig.3f). Substituting the Eqs. (4) and (8) in the Eq. (1), the QIF-E$_{dfl}$ is obtained, and is provided by the expression:

10 $$\begin{aligned} \frac{dV_{m}(t)}{dt}= & {} \frac{(V_{m}(t)-V_{rest})(V_{m}(t)-V_{{thresh}})}{{R_{dfl}} {C_{dfl}}(V_{{thresh}}-V_{rest})} - \frac{\rho _{ext} I_{eph}(t)}{4\pi {R_{dfl} C_{dfl}} r}+ \frac{I_{0}}{C_{dfl}(1-h)} \end{aligned}$$

11 $$\begin{aligned} \frac{dV_{m}(t)}{dt}= & {} \underbrace{\frac{(V_{m}(t)-V_{rest})(V_{m}(t)-V_{{thresh}}){(1-b)}}{{R_{m}C_{m}} (V_{{thresh}}-V_{rest}){(1-h)}}}_{{\textbf {Quadratic term}}} - \underbrace{\frac{\rho _{ext} I_{eph}(t){(1-b)}}{4\pi {R_{m} C_{m}} r {(1-h)}}}_{{\textbf {Ephaptic term}}} + \underbrace{\frac{I_{0}}{C_{m}{(1-h)}}}_{{\textbf {Synaptic term}}} \end{aligned}$$

The QIF-E$_{dfl}$ MatLab code is available in the Supplementary Material.

### Spike field coherence - SFC

The SFC is a tool to measure the synchrony intensity, between a stimulus signal (Fig.1d in red) and a spike train (Fig. 1d in black), based on Spike Triggered Average (STA) analyses^45,47^. Thus, the SFC provides, given an external stimulus frequency (LFP), the entrainment preference to neuronal spikes. Through this procedure, empirical study^45^ and neuronal modeling^41^ estimated the ephaptic communication intensity.

The Spike Triggered Average (STA) is a tool to calculate the mean field profile to occur a spike in a neuron related to the preference input stimulus phase^48^. The STA associate two signals: an input signal and the membrane potential response. To obtain the STA one should take parts of the input stimulus interval, $l_{i}$, around the spikes instants in the neuron signal^49^. This intervals, $l_{i}$, are chosen with a time window defined by simulation conditions. In the present work, the temporal window assumed to obtain the STA is $\frac{1}{f}$ for *f* the stimuli frequency. Therefore, the STA describes the stimulus mean field where associated to a high spike occurrence probability.

**Figure 4:**
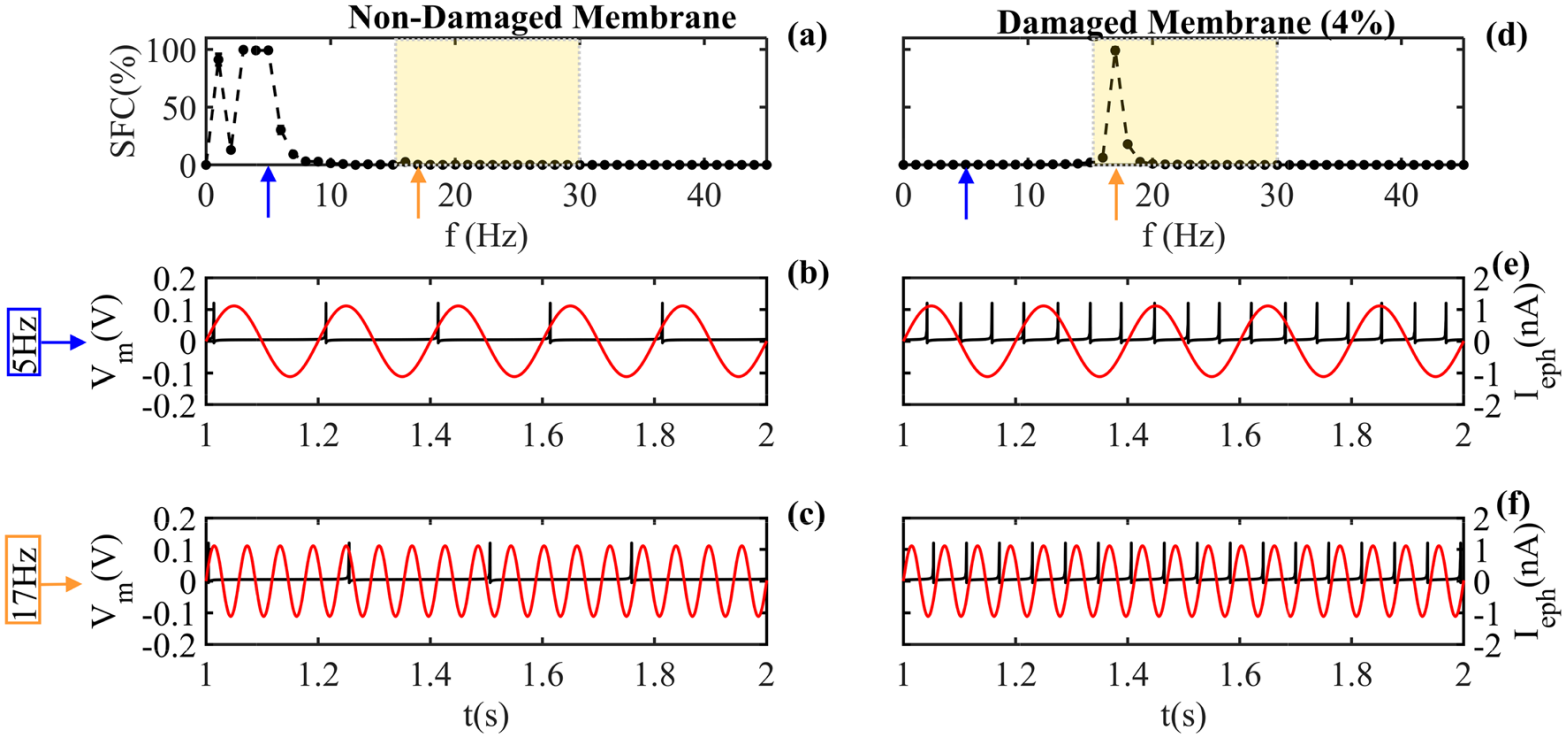
**SFC of QIF-E**$_{dfl}$ and temporal series. Panels (**a**–**c**) correspond to a healthy membrane (b = h = 0.00), while panels (**d**–**f**) correspond to a degenerate membrane (b = h = 0.04). Blue and orange arrows indicate frequencies (**b**), (**e**) - 5Hz and (**c**), (**f**) -17Hz. Two signals are indicated in these panels, the simulated QIF-E$_{dfl}$ (spikes in black) and the input LFP (sine curve in red). In the healthy case, the spikes synchronize with the external LFP phase signal (red) at f=5Hz, but not at f=17Hz, resulting in a high SFC value for low frequencies (blue arrow) in figure (**a**). The opposite occurs in the damaged membrane case, here the spikes synchronize with the external LFP phase signal at f=17Hz, but not at f=5Hz, resulting in a high SFC value for higher frequencies (orange arrow) in figure (**d**).

Finally, the spike field coherence (SFC) is a tool that measures how strong is the synchronization between a stimuli signal phase and a spike train, based in the STA analysis^45,47^. The calculation to SFC is performed by the expression^50^ $SFC = \frac{\Psi (STA)}{\frac{1}{n}\sum ^{n}_{i=1}\Psi (l_{i})}$, where $\Psi ()$ is the power spectrum, STA is the Spike Triggered Average. Therefore, the SFC it is defined between 0 (without signal synchrony) and 1 (totally synchronous signal) [see Fig. 4a and (d)]^47^. The Fig. 4a,d, exhibit the SFC for different frequencies of external stimulus, from 0 to 45Hz. In (a), we observe the preference frequency below 10 Hz (blue arrow in 5Hz corresponds to the time series observed in (b)). The yellow arrow at 17Hz indicated a 4% preference frequency for a damaged membrane. We depicted the time series for 5Hz in (b,c) and the time series for 17Hz in (e,f). We performed the SFC calculus in MatLab.

## Results

To show the results of the synchronization between the LFP-phase and the spikes (model response), we use the SFC tools for the cases without damage, seen in panels (a) of each figure, as well as the results with different levels of damage in the interval from 4% (0.04) in panels (b) to 20% (0.20), in panels (f), with a step of 4% (0.04). For simulations, we used the ephaptic model with damage QIF-E$_{dfl}$. For damage above 20% and accurate steps, see Supplementary Material.

**Figure 5:**
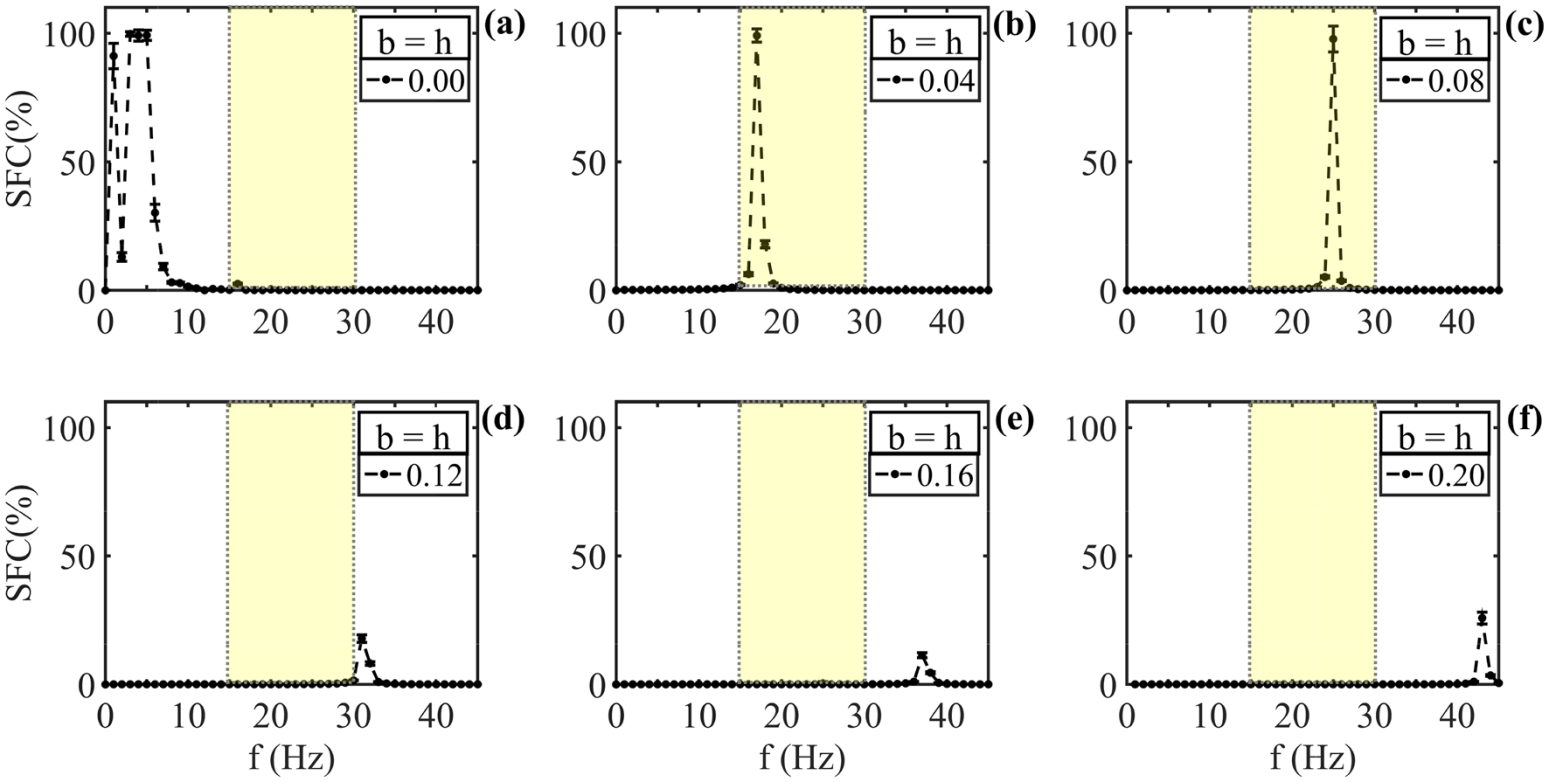
Ephaptic model response for an LFP with both: ionic channels and the membrane bilayer damages. (**a**) SFC for the QIF-E$_{dfl}$ model without damage (b=h=0.00). Note an entrainment preference in the frequency bands below of 10Hz. (**b**) SFC results for damage membrane of 4% (0.04). A shift occurs in the ephapticty frequency preference between 15 Hz and 19 Hz. (**c**) SFC with 8% of damage and ephaptic preference in the frequency band between 23 and 27 Hz. (**d**) SFC with damage to 12% and entrainment preference shift to the frequency band of 30 to 34 Hz. (**e**) SFC for damage of 16% and ephaptic entrainment in the frequency band of 36–39 Hz. (**f**) SFC with damage of 20% and preference in frequency band between 42 and 45 Hz. The yellow shadow region is defined as $\beta$ the oscillation band.

The Fig. 5 shows the SFC for the equals damage values, i.e., $b = h$. In 5a there is no damage in the simulation (electrophysiology standard, $b = h = 0.00$) and consequently an entrainment preference in the frequency band stay below 10 Hz to the ephapticity. Note that in (b), for damage to 4% in the both parameters, the ephaptic entrainment shifted to an oscillation range in the $\beta$ band (yellow shaded region); in (c) the damage is 8% and observe an ephaptic entrainment in frequency band of 23–27 Hz, also in the $\beta$ band. In the (d) panel, the damage is 12% and the entrainment occurs in the $\gamma$ band. It is interesting to note that the SFC intensity decrease with this damage intensity. In (e) the damage is 16% and the entrainment band occur around of 37Hz. Finally, in (f) the damage is 20% and the ephaptic entrainment is close to 42Hz.

For values of neuronal degeneration (damage) above 0.20, do not observed peak of entrainment in the study band frequency (0 to 45 Hz) (See Supplementary Material).

**Figure 6:**
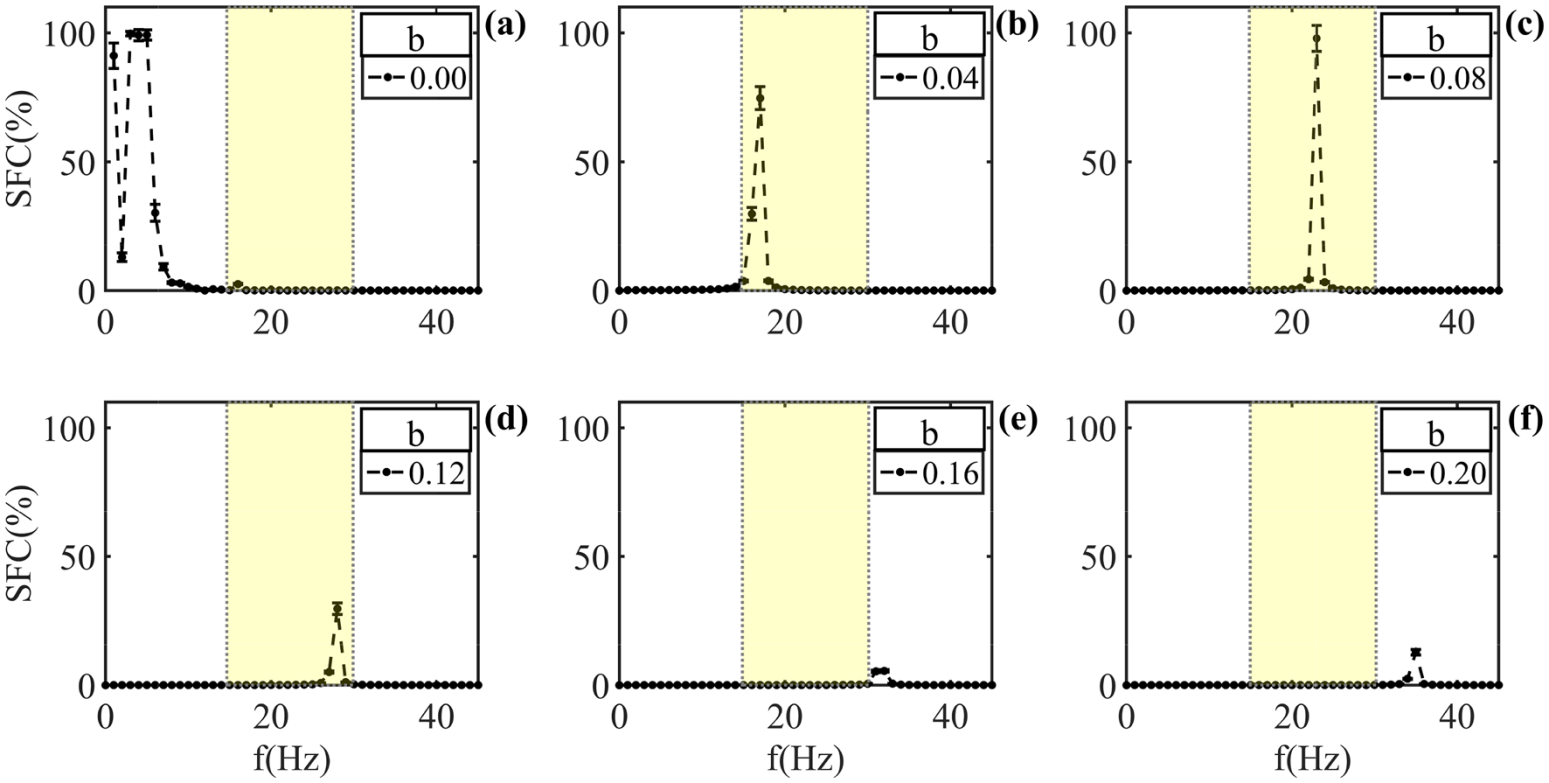
Ephaptic model response to LFP with neuronal damage in ionic channels only. *b* values between 0.00 and 0.20, step 0.02 chose between each simulation. In (**a**) the SFC shown without damage, equal to Fig.5(**a**). (**b**) SFC to $b = 4\%$. Here occur an ephaptic entrainment displacement for the band frequency in range of 15–19 Hz, similar result was shows in 5(**b**). (**c**) SFC of $b = 8\%$ and a frequency preference in the band of 21 to 25Hz. In (**d**) the SFC for $b = 12\%$ shows a frequency preference between 26 and 29 Hz. (**e**) SFC with $b = 16\%$ and frequency preference for entrainment in 30 to 33 Hz band. (**f**) SFC for $b = 20\%$ and band preference between 33 and 36 Hz. The yellow shadow region is defined as $\beta$ the oscillation band.

The Fig. 6 shows the SFC for damage values only in the resistance (*b*) lying between 0 and 20%. In Fig.6a the simulation present physiologic standard, and an ephaptic entrainment band preference below 10 Hz, as observed in Fig. 5a. Sequentially, with $b \in [2,20]\%$ and $h = 0.00$, observed a migration behavior in the ephaptic entrainment again, to high frequency bands ($f > 10Hz$) when compared with $b = 0.00$. Thus, in (b) is shown damage to 4% and frequency band preference around 16 Hz. In (c) that damage is 8% and the oscillation preference is round about to 22Hz; in (d), with the damage to 12% and ephaptic entrainment preference between 26 and 29 Hz. (e) shows damage in 16%, and finally in (f) the damage is 20%. Note that from the damage to 16%, the coupling intensity decrease and lying out of $\beta$ frequency band ([13–30] Hz). In this figure, here is a similarity with Fig. 5 in terms of frequency preference changes. The similarity was observed include between the Figs. 5a and 6a in the frequency preference, just as between Figs. 5b and 6b.

As from $b = 12\%$, is observed a decrease of intensity in the model ephaptic entrainment.

**Figure 7:**
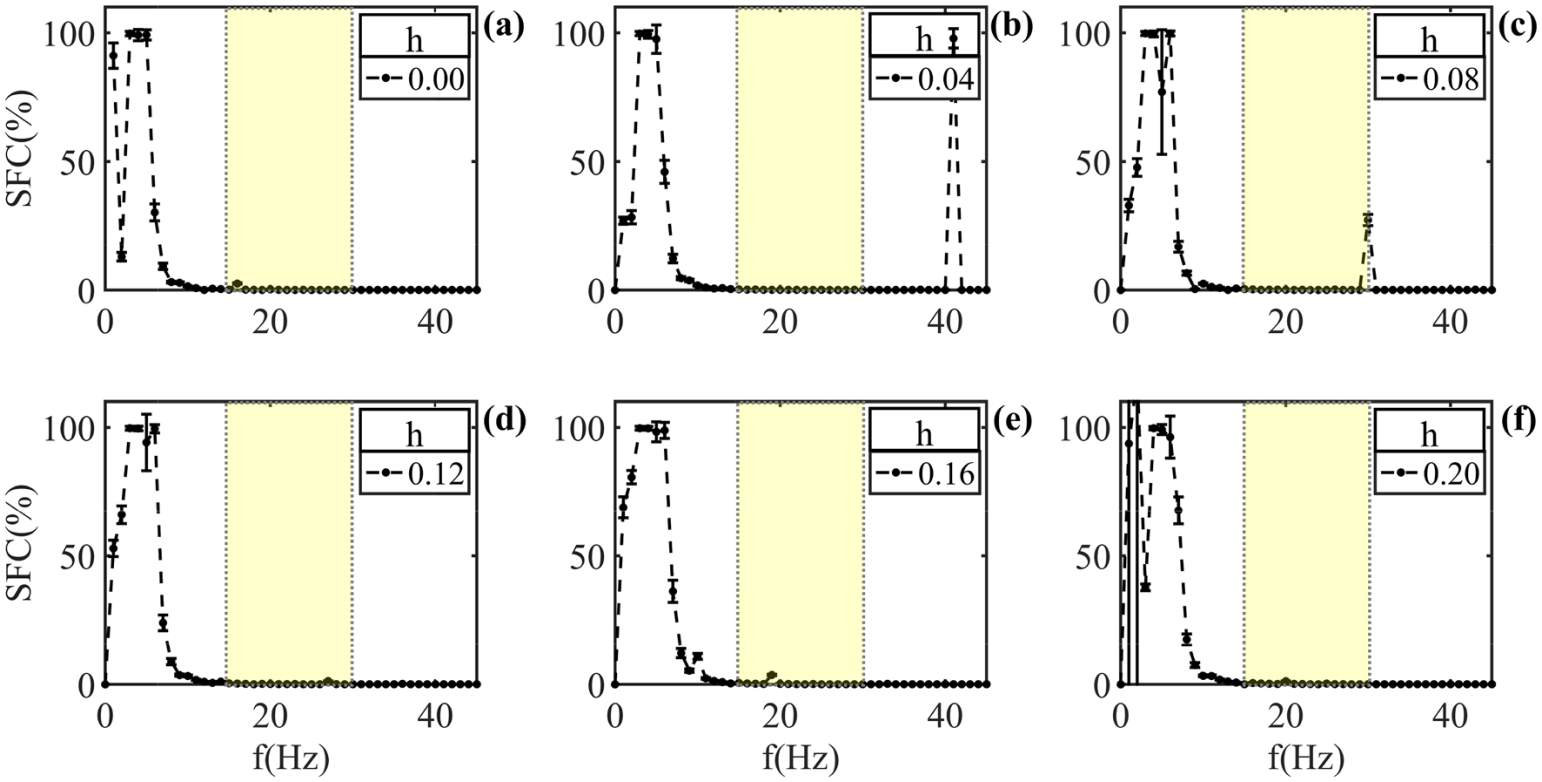
Ephaptic model response for an LFP, with damage in membrane bilayer only. *h* values between 0.00 and 0.20, step by 0.02, and *b* fixed. (**a**) SFC for QIF-E$_{dfl}$ no damaged. In (**b**) shown SFC for $h = 4\%$. Note entrainment preference retained in the low frequency ($f < 10Hz$), and SFC presents a maximum in the band above 40Hz. (**c**) The SFC with $h = 8\%$ shows a preference of ephapticity also in low frequencies. A new peak, smaller, is observed in the band of 29 to 31 Hz. In (**d**–**f**) the SFC with 12, 16 and $20\%$ of damage, respectively, the behavior is similar to the shown in panel (**a**).

Figure 7 shown SFC values to changes in the capacitance degeneration, with fixed *b*. As noted in Figs. 5a and 6a, the Fig. 7a shows intense SFC in the low frequencies ($f < 10Hz$). The difference in the Fig. 7b–f, compared to previous figures, is that the maximum value in the ephaptic entrainment not shift to the high frequencies. However, note that for $h = 0.04$ and $h = 0.08$ high frequencies peaks occurs together with the low frequencies maximum. Finally, in the Fig. 7f is possible to note that here significant dispersion in relation to the mean for a few values of low frequency band.

## Discussion

The present work shows the proposal of the membrane, (Fig.2a) as a set of unitary elements (Fig.2b), where each unit is equivalent either or to an ion channel (Fig.2c), or to a phospholipid structure (Fig.2d). The unitary elements that form the membrane are associated with electrical components: ion channels as electrical resistance (Fig.2e) and phospholipids as capacitors (Fig.2f). Thereby, we propose two damage parameters that mimic the resistance impairment (*b*), and the capacitor’s impairment (*h*). Sequentially, we estimated, using the QIF-E^41^ hybrid model, how ephaptic communication is affected by dysfunctions in neuronal membrane electrophysiology (Fig.3). As a result, for a simulated neuronal membrane damage levels, we obtained different preference frequency bands (via SFC—Fig. 4) for ephaptic entrainment. Furthermore, with the increase of the impairment in the generic ion channels and in the neuronal membrane, the maximum value of ephaptic entrainment is shifted to higher frequencies (f > 10Hz), as observed in Fig. 5.

Taking into account that the same pattern of deterioration happens in the phospholipidic bilayer (capacitance) and in ion channels (resistance), we adopted equal absolute values for both damages in the simulations, i.e., $b=h$. In the Figs. 5, 6 and 7 (a)’s, for non-damage membrane, the SFC presents high intensities for low frequency values ($f<10$Hz) due to the characteristics of ephaptic communication, since the neural tissue works as a low-pass filter, as indicated in empirically^4,51,52^ and theoretically^41^ studies. In Fig. 5b and (c), the intensity of the SFC preference (peak value) remains high and within the range $\beta$ band (13–30 Hz), with impairment between 2% and 10% ($b=h=0.02$–0.1). As damage increases, there is a shift in the frequency preference of ephaptic communication and also a reduction in strength entrainment, see panels (d), (e) and (f). This decrease in SFC intensity is due to the above-mentioned low-pass filter characteristic of neural tissue, which reduces the amplitude of high-frequency signals^4,51,52^. For damage higher to 20%, ephaptic communication does not occur in analyzed frequency band. Fact observed by the absence of preference in the frequency band provide in the SFC (flat) (See Supplementary Material).

The ephaptic entrainment simulation demonstrates frequency preferences that are determined by the electrophysiological characteristics of the membrane. These characteristics can be modified by dysfunctional oligomers, such as $\alpha$-synuclein, which are found in the cellular environment, as outlined in other studies^15,18,22–26^. In this regard, our study unveils a potential relationship between electrophysiological impairment of the neuronal membrane and alteration in the ephaptic mechanism linked to degenerative disorders. For instance, in AD, there is a lack of synchronization in the $\alpha$ (8 - 13 Hz) and $\beta$ (13 - 30 Hz) frequency bands, and a pronounced synchronization in the low frequency bands ($f < 8$ Hz)^27,31,32^. On the other hand, an escalation in the intensity of the $\beta$ band has been observed in patients with Parkinson’s Disease^28–30^. Once the frequencies observed in those disorders reach the upper threshold of 35 Hz, the model was executed utilizing LFP within the range of 0–45 Hz.

In order to evaluate the influence of altering neuronal membrane resistance (*b*) on frequency preference for ephaptic entrainment, we established the value of $h = 0$ (indicating no phospholipid damage) and performed simulations of QIF-E$_{dfl}$ with *b* ranging from 0 to 20%. Figure 6 illustrates the changing in the *b* parameter and a corresponding alteration pattern in frequency preference, akin to the depiction exhibited in Fig. 5. However, it is noteworthy that, at an equivalent level of damage, the frequency preference (in the LFP) is shifted into smaller values in comparison to those observed for the case when $b = h$. In general, the alteration in frequency preference, at which the maximum ephaptic coupling takes place, is associated with the dynamics of ion channels, specifically their opening and closing mechanisms. This observation is reasonable, given that during the membrane potential variation, ion channels generate currents towards the extracellular medium and thereby produce ephaptic fields^40,43^.

Finally, the ephaptic response was examined by considering the reduction in capacitance of the neuronal membrane with no damage to the ion channels, as illustrated in Fig. 7. In this scenario, it is observed that the maximum value of ephaptic coupling persists at low frequencies ($f < 10$ Hz), as depicted in Fig. 7b–f. However, the standard-deviation values associated with each SFC value increases. This suggests that when considering alterations solely in the phospholipid layers, there is no notable modification in the preferred frequency range of maximum efficacy compared to the frequency preference observed in the healthy condition [see panel (a)]. This evaluation was conducted with damage levels of up to 20%. Modifications in both the phospholipid layers and cholesteric content within the neuronal membrane might be linked to heightened noise in ephaptic communication. This association has been previously indicated in a prior study^41^. The increase in background noise does not change the phase or the frequency of the ephaptic communication, but it can change the intensity of the ephaptic entrainment, which is linked to an increasing of the SFC error bars.

The outcomes of the current study intimate that the detriment to the electrophysiological attributes of the neuronal membrane exhibit a comprehensive influence on the ephaptic communication, engendering a discernible alteration in its interactive dynamics^23,24,53–55^. Hence, it can be posited that the ephaptic communication assumes a pivotal position in shaping the dynamic framework of the Central Nervous System, though the exact nature of this role continues to elude our understanding. Moreover, given its potential implications on neuronal efficacy, the intricate interplay of ephaptic mechanisms within the central neural networks warrants further investigation and exploration^3,4,9^. Indeed, the dysfunctionality that arises within neuronal membranes, particularly in the ionic channels, as a result of misfolded proteins, has been linked to an elevation in neuronal activity within specific oscillation bands. This phenomenon has been extensively documented in the scientific literature and is prominently observed in degenerative conditions such as Alzheimer’s disease^12,15,27^, Parkinson’s disease^28,29,56^, and Lewy body dementia^27,31,32^. Understanding the intricacies of ephaptic communication in the context of these degenerative disorders can assume paramount importance in advancing our knowledge of their pathophysiology and potentially uncovering novel therapeutic avenues.

## Supplementary Information

Supplementary Information.

## Data Availability

The datasets generated during and/or analyzed during the current study are available from the corresponding author on reasonable request.
