# Supplementary material for: Electrophysiological damage to neuronal membrane alters ephaptic entrainment
Source: Sci Rep. 2023 Jul 24;13:11974. doi: 10.1038/s41598-023-38738-x (PMC10366241; doi:10.1038/s41598-023-38738-x)
Supplement: Supplementary file 1 — Supplementary Information. [file 41598_2023_38738_MOESM1_ESM.pdf]

Supplementary Information

Title: Electrophysiological damage to the neuronal membrane alters ephaptic communication.

Authors:
Gabriel Moreno Cunha,
Marcelo M. S. Lima,
Gilberto Corso,
*Gustavo Zampier Dos Santos Lima.

1 Spike Field Coherence for different impairment level

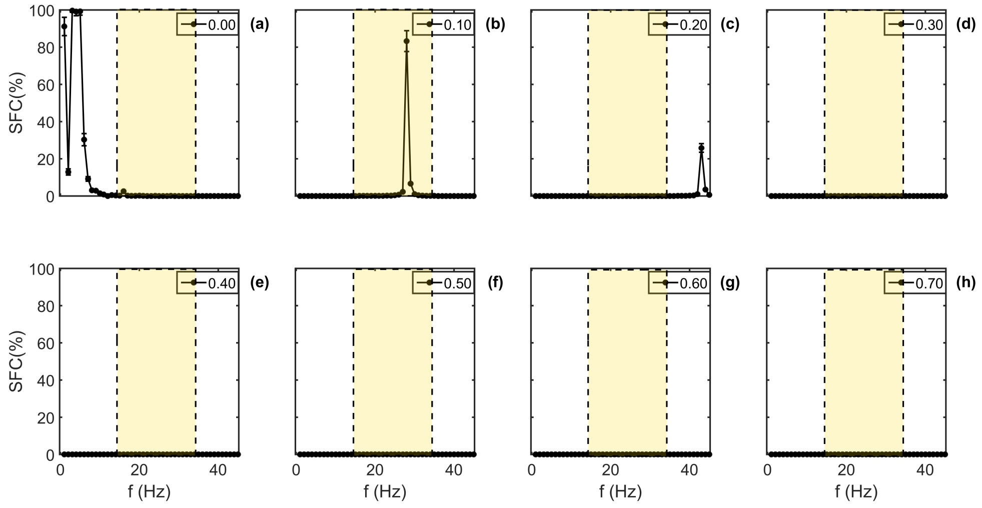

Figura 1: Impairment level for $b = h$, with more step detail.

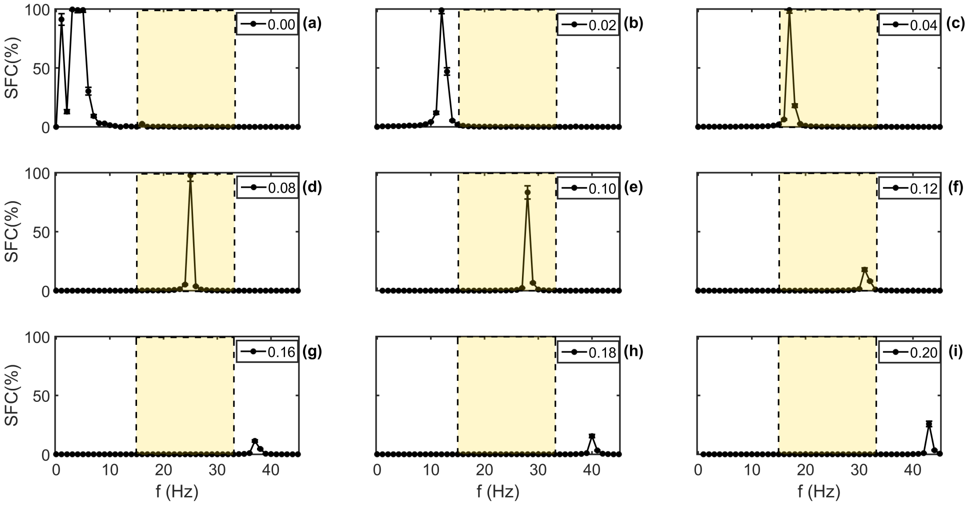

Figura 2: Impairment level for $b = h$, with more step detail.

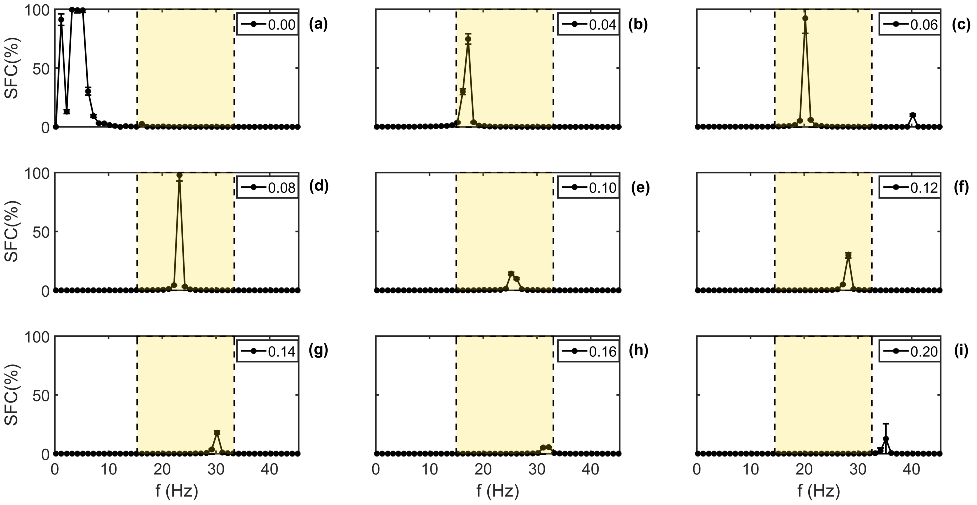

Figura 3: Impairment level for $b > h = 0$, with more step detail.

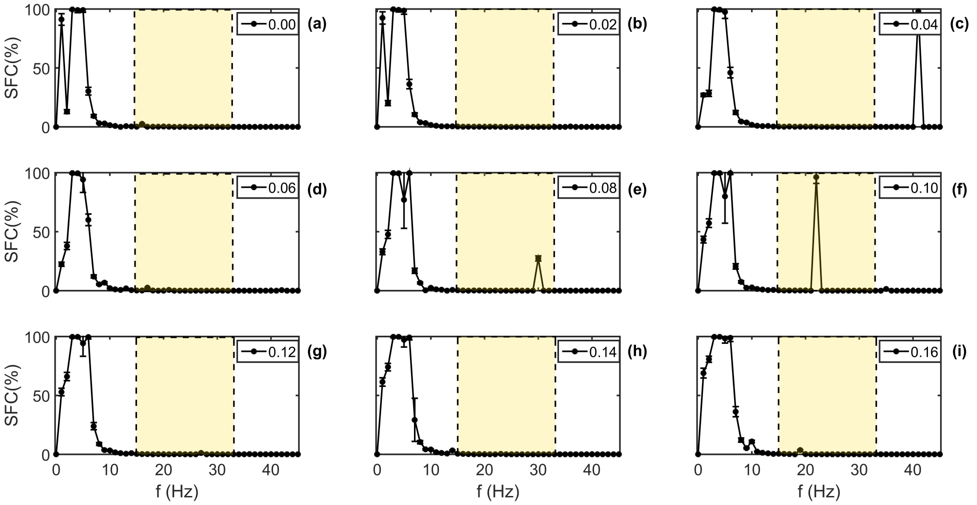

Figura 4: Impairment level for $h > b = 0$, with more step detail.

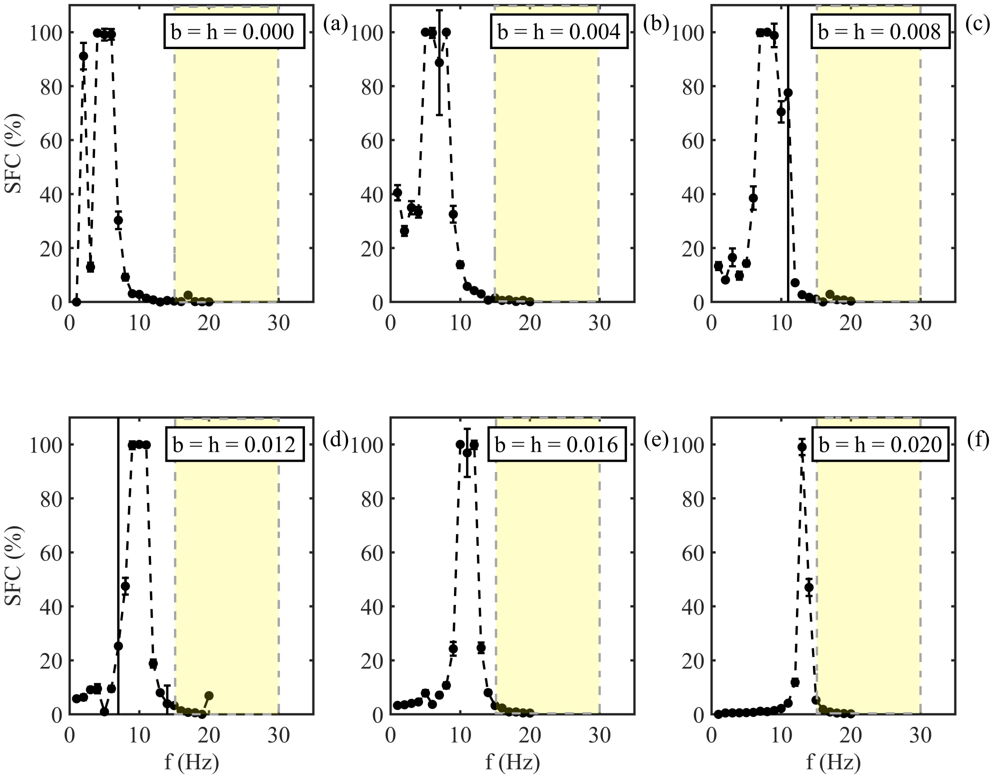

Figura 5: Impairment level for $b = h$, between 0.000 and 0.020, step by 0.004.

2 QIF-E$_{dfl}$ code

```
% Quadratic integrate-and-fire ephaptic model, powered by Gabriel Moreno
% Cunha. Universidade Federal do Rio Grande do Norte (UFRN), Natal-RN. 2021
% Modelo integra e dispara quadratico efatico, desenvolvido por Gabriel
% Moreno Cunha. Universidade Federal do Rio Grande do Norte (UFRN),
% Natal-RN. 2021
clear all

%% Escale Variables - Variaveis de Escala:
percent = 10^(-2);
kilo = 10^(3);
mili = 10^(-3);
micro = mili^2;
nano = micro * mili;
pico = nano * mili;

%% Simulation Varaibles - Variaveis de simulacao:
```

```
sample = 10000;                         %Sample rate - Taxa amostral
tempo = 20 ;                            %Time (seg) - Tempo (seg)
pto = tempo * sample;                   %Number of points - Numero de pontos
pass = tempo / pto;                     %Pass - Passo de integracao

%% Model Variables - Variaveis do Modelo:
freq = 1;                               %External Frequency - Frequencia Externa
snr = 160;                              %SNR (dB) - SNR (dB)
Amp = 5 * nano;                         %External Current Amplitude - Amplitude da Corrente Externa
V_m = [];                               %Membrane Potential - Potencial de Membrana
I_ext = [];                             %Extenal Current - Corrente Externa
I_int = 2.506 * micro;                  %I_0 Current [I_0 = 0 subthreshold;I_0 ~= 0 suprathreshold] -
                                        %Corrente I_0 [I_0 = 0 sublimiar;I_0 ~= 0 supralimiar]

%% Model Constants - Constantes do Modelo:
b = 0.1;                                %Resistance Impairment - Degeneracao da resistencia
h = 0.1;                                %Capacitance Impairment - Degeneracao da capacitancia
V_rest = -65 * mili;                    %Rest - Potencial de Repouso
V_lim = -55 * mili;                     %Excitation Limit - Limiar de Excitacao
peak = 55 * mili;                       %Peak Value - Valor do Pico
Resis_m = (1*kilo)/(1-b);               %Membrane Resistance(Ohm.cm^2)- Resisitencia da membrana(Ohm.cm^2)
Capac_m = 2 * micro * (1-h);            %Membrane Capacitance(F/cm^2)- Capacitancia da Membrana(F/cm^2)
r = 50 * micro;                         %Distance between Neuron x Electrode (\mu m) - Distancia entre o Eletrodo e o Neuronio(\mu m)
resis = 3.5 ;                           %Extracelullar Resistivite(Ohm.M) - Resistividade do meio extracelular(Ohm.M)
c = 4 * pi;                             %Constant - Constante
C1 = Resis_m * (V_lim - V_rest);        %QIF Model Constant - Constante do modelo IDQ
C2 = resis /(c * r);                    %Constante - Constante

%% ----------------------------------------------------------------
%--------------------Simulation Area-------------------------
%-------------------Area de Simulacao------------------------
%----------------------------------------------------------------

V_m(1) = V_rest;                        %Initial
```

```
Condition - Condicao inicial

I_ext = Amp * sin(2 * pi * freq * (pass:pass:tempo));                          % External Current - Corrente Externa;
I_ext = awgn(I_ext,snr,'measured');                                            %Add White Gaussian Noise - Adicao do ruido branco gaussiano
V_e = I_ext .* C2;                                                             % Ephaptic Potential - Potencial Efatico
I_m = V_e./Resis_m;                                                            % Ephaptic Transmembrane Currente - Corrente Efatica Transmembrana

for i = 1:1:pto-1
  V_m(i+1) = V_m(i) + (((V_m(i)-V_rest)*(V_m(i)-V_lim)/C1) + I_int - I_m(i)) * pass / (Capac_m); %Discrete Model Equation - Equacao Discreta do Modelo
   if V_m(i+1) >= peak
       V_m(i) = peak;
       V_m(i+1) = V_rest-(5*mili);                                             % Reset Model after spike - Resetando o Modelo apos o PA
   end
end

V_r = V_m - V_rest;                                                            %Put V_m in zero - Coloca V_m em zero
x=pass:pass:length(V_r)/sample;                                                %Better resolution - Melhor resolucao



%% -------------------------------------------------------------
%---------------------Graphic Area-----------------------------
%----------------------Area Grafica----------------------------
%--------------------------------------------------------------

figure1 = figure('Color',[1 1 1]);

%% ------ Plot Membrane Potential - Grafico do Potencial de Membrana

subplot(211)
ax = gca;
plot(x,V_r,'.-','color','g');
xlim([10 10+2/freq])
ylim([min(V_r) max(V_r)])
ylabel('V_{m} - V_{rest}(V)','FontSize',16);
legend({'Membrane Potential', 'color', 'g'},'Location', 'northeast','FontSize',16);
ax.FontSize = 16;

%% ------- Plot LFP - Grafico do LFP

subplot(212)
ax = gca;
plot(x,V_e,'.-','color','r');
xlim([10 10+2/freq])
ylim([min(V_e) max(V_e)+10^(-4)])
xlabel('Time(s)','FontSize',16);
ylabel('V_{e}(V)','FontSize',16);
legend({'Local Field Potential', 'color', 'b'},'Location', 'northeast','FontSize',16);
ax.FontSize = 16;
```

```
%%
clear all
```
